# Fear of Progression, Anxiety, and Depression in Patients With Advanced Melanoma in the COVID-19 and Post-COVID-19 Era

**DOI:** 10.3389/fpsyt.2022.880978

**Published:** 2022-04-26

**Authors:** Xiaowen Wang, Min Li, Qiong Shi, Hongchen Ji, Shengnan Kong, Lei Zhu, Hong-Mei Zhang

**Affiliations:** ^1^Department of Clinical Oncology, Xijing Hospital, Fourth Military Medical University, Xi’an, China; ^2^Department of Neurosurgery, Shaanxi Provincial People’s Hospital, Xi’an, China; ^3^Department of Dermatology, Xijing Hospital, Fourth Military Medical University, Xi’an, China; ^4^School of Psychology, Shaanxi Normal University, Xi’an, China

**Keywords:** fear of progression, anxiety, depression, melanoma, COVID-19

## Abstract

**Background:**

The novel coronavirus disease 2019 (COVID-19) pandemic causes great disruption to cancer care services, which might bring about psychological problems and further lower both physical and mental life quality in cancer patients. Until now, very few studies focused on the psychological distress of patients with advanced melanoma before or during the epidemic. This study aimed to elucidate the fear of progression (FoP), anxiety, depression, and related independent predictors in patients with advanced melanoma during the COVID-19 outbreak.

**Methods:**

Two hundred and seventy-three patients with unresectable stage III or metastatic melanoma were recruited from February 2020 to November 2021, and completed the Fear of Progression Questionnaire-Short Form (FoP-Q-SF), State Trait Anxiety Inventory (STAI-6), and Patient Health Questionnaire (PHQ-9).

**Results:**

One hundred and seventy-four (64.7%) patients experienced heighted FoP (FoP-Q-SF: 39.9 ± 11.0), 198 (72.5%) patients reported elevated anxiety (STAI-6: 13.1 ± 3.0), and 62 (22.7%) patients had increased depression (PHQ-9: 6.4 ± 6.1). In multivariate analysis, illness duration (*OR* = 0.987 for FoP; *OR* = 0.984 for depression), cancer stage (*OR* = 14.394 for anxiety) and disease progression (*OR* = 1.960 for FoP; *OR* = 23.235 for anxiety; *OR* = 1.930 for depression) were independent predictors for FoP, anxiety or depression. Additionally, the high levels of FoP, anxiety and depression were significantly positive correlated with each other (*r* = 0.466 for FoP and anxiety; *r* = 0.382 for FoP and depression; *r* = 0.309 for anxiety and depression).

**Conclusion:**

Our study indicates that FoP, anxiety and depression are persisting among patients with advanced melanoma in the COVID-19 and post-COVID-19 era. Effective psycho-oncological interventions are needed for melanoma patients with psychological distress during the ongoing COVID-19 pandemic.

## Introduction

The novel coronavirus disease 2019 (COVID-19) pandemic, caused by severe acute respiratory syndrome coronavirus 2 (SARS-CoV-2) (1), has occurred in 237 countries, areas or territories and turned into a global public health crisis (2). Globally, as of 17 December 2021, there have been 271,963,258 confirmed cases of COVID-19, including 5,331,019 deaths (2). The COVID-19 pandemic continues to create significant challenges globally (3), not only to effectively handle the COVID-19 pandemic but also to manage other diseases especially cancer (4). Previous studies suggest that cancer patients might be particularly susceptible to COVID-19 and have a poorer prognosis because of their immunosuppressive condition caused by the cancer itself and anticancer treatments, such as surgery, chemotherapy, radiotherapy, targeted therapy, or immunotherapy (5). A major consideration of cancer care is to balance the need of cancer management against the risk of patient exposure and infection in the face of the COVID-19 pandemic (4). In order to reduce or avoid cross infection, many hospitals have taken mandatory actions to limit outpatient visits and inpatient admissions (6, 7). The pandemic causes great disruption to the full spectrum of medical cancer care services, including cancer diagnoses, treatments and follow-up (8).

Melanoma is a malignant neuroendocrine tumor of neural crest and mainly occurs in skin and mucosa. The morbidity and mortality of melanoma has increased dramatically around the world (9). There were 324,635 new cases of melanoma, the equivalent of about 889 new cases each day worldwide in 2020 (9). An estimated 57,043 people died from melanoma worldwide in 2020, corresponding to almost 156 deaths per day (9). The median overall survival time of patients with metastatic melanoma is only six to 8 months (9). Immune checkpoint inhibitors and targeted therapy have improved survival outcomes of melanoma patients, however, the prognosis of patients with advanced melanoma remains unoptimistic (10, 11). Recent researches demonstrate the unfavorable effects of COVID-19 on advanced melanoma care (12–15). During the COVID-19 pandemic, the diagnose, start of systemic treatment and treatment courses for advanced melanoma were frequently postponed (12). Attentionally, along with the epidemic, patients were diagnosed with poorer tumor characteristics (12). Forced delays or interruptions of cancer management might increase the risk of cancer deterioration and therefore bring about physical and psychological problems in patients with melanoma.

Previous reviews denote that about 30% of melanoma patient suffered from heightened psychological distress during the time of diagnosis and treatment (16, 17). Fear of cancer recurrence, anxiety and depression are highly common psychological symptoms (18, 19). Fear of cancer recurrence is defined as worry, or concern about cancer relapse or fear of progression (FoP), with prevalence rates of 31–52% (18). Anxiety is characterized by an emotional state consisting of feelings of apprehension and tension and arousal of the autonomic nervous system, with prevalence rates of 9.8–19% (19, 20). Depression includes a depressed mood and/or loss of interest or pleasure in normal activities, with additional symptoms including worthlessness, guilt, concentration problems and changes in appetite, energy and sleep, with prevalence rates of 8–24.6% (19, 21). Untreated negative psychological symptoms could further lower both physical and mental life quality of cancer patients (22), which might be worse in those patients who undergo anti-cancer treatments.

Given the strong effect of COVID-19 pandemic on cancer care, cancer patients are more susceptible to emotional attack without enough attention and adequate psychological support. Before the epidemic, very few studies focused on the mental state and independent predictors in patients with advanced melanoma. Currently, little is known about the psychological distress in melanoma patients during the public crisis. This cross-sectional study focused on the negative psychological symptoms of patients with advanced melanoma in the COVID-19 and post-COVID-19 era. We assessed the prevalence of FoP, anxiety and depression in patients with advanced melanoma. Furthermore, we explored the association of demographic and illness-related factors with the levels of FoP, anxiety, and depression. Third, we examined the correlationship between FoP, anxiety, and depression in melanoma patients. The present study is needed to provide a basis for psychological intervention.

## Methods

### Sample and Procedure

**FIGURE 1:**
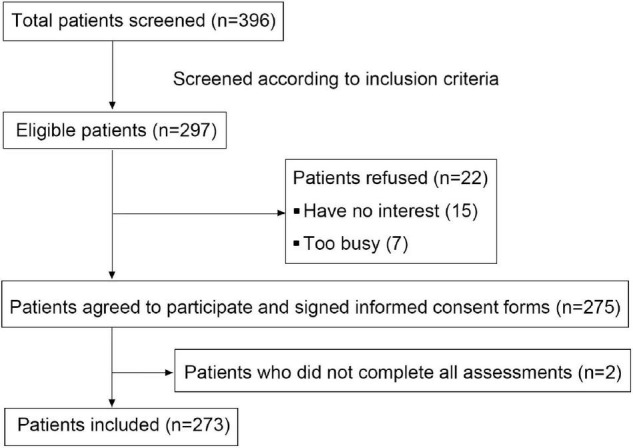
Recruitment flowchart.

To investigate the influence of COVID-19 outbreak on the mental state of patients with advanced melanoma, a cross-sectional single center study was performed in a general Hospital named Xijing hospital of the Fourth Military Medical University. Eligible melanoma patients were recruited consecutively from February 2020 to November 2021. Patients were eligible if they were diagnosed with advanced melanoma. Patients were excluded if they had severe physical impairment (with an Eastern Cooperative Oncology Group performance-status score greater than or equal to 2) and/or severe cognitive impairment. Patient recruitment flowchart is shown in Figure 1. In total of 396 patients, 297 patients were eligible and 275 patients agreed and signed an informed consent. The reasons for refusal were: not interested in the study (15 patients) and too busy (7 patients). Finally, 273 patients completed the questionnaires. The response rate was 91.9%. No patients received a diagnosis of COVID-19 infection in the study population confirmed by SARS-CoV-2 nucleic acid tests. The study was approved by the Committee for Ethics in Medical Investigations of the Forth Military Medical University and was conducted according to the Declaration of Helsinki Principles. All patients signed an informed consent provided electronically prior to registration, and eligible patients completed the questionnaires through online platform.^1^

### Population Characteristics

Socio-demographic characteristics (e.g., age, gender, educational level, marital status, and salary) were obtained through a self-report questionnaire. The clinical characteristics (e.g., diagnosis time, cancer stage, cancer recurrence or progression, and current treatment) were collected from patients and confirmed through medical record of hospital. Educational level was classified into three levels: low (primary schooling and lower vocational education), middle (secondary schooling and middle vocational education) and high (university education and higher vocational education).

### Fear of Progression

FoP was measured with the Fear of Progression Questionnaire-Short Form (FoP-Q-SF) (23). Each item was rated on a 5-point Likert scale from 1 (never) to 5 (very often). The total score ranges from 12 to 60. A cut-off score of ≥34 indicates clinical FoP. The Chinese version of scale has good reliability and validity in Chinese cancer patients (Cronbach α = 0.883) (24).

### Anxiety

Anxiety was measured with the six-item short form of State Trait Anxiety Inventory (STAI-6) (25). Questions were answered on a 4-point Likert scale from 1 (not at all) to 4 (very much). Total scores range from 6 to 24, with higher scores indicating greater anxiety. A score ≥ 12 indicates significant anxiety. This scale has good reliability and validity in cancer patients (Cronbach α: 0.77–0.83) (26).

### Depression

Depressive symptoms were measured with the nine-item short form of Patient Health Questionnaire (PHQ-9) (27). Each item was answered on a 4-point Likert scale from 0 (never) to 3 (nearly every day). Total scores ranged from 0 to 27, with cut-off scores of 10 or higher for a diagnosis of major depression. Previous research in cancer patients has shown good reliability and validity (Cronbach α: 0.89–0.92) (28).

### Statistical Analysis

Statistical analyses were two-sided and performed by SPSS software (Version 26.0, SPSS Inc.). First, test of normality was performed by one-sample Kolmogorov-Smirnov test. Descriptive statistics were used to describe the demographic and clinical characteristics and psychological symptoms of study population, defined as mean with standard deviation (SD) or frequency with percentage. Second, continuous variables were not normally distributed, univariate analysis for factors associated with FoP, anxiety or depression was performed using Mann–Whitney *U* tests or Kruskal–Wallis tests. Third, independent predictors of high FoP, anxiety, or depression were tested in multivariate logistic regression. Finally, correlation was used to detect the relationship between FoP, anxiety, and depression by Spearman’s correlation. Statistical significance was defined as *P* < 0.05.

## Results

### Study Population

A total of 273 patients with advanced melanoma were enrolled in the present study. The socio-demographic and medical characteristics of participants are shown in Table 1. The mean age was 56.9 years (*SD* = 13.2), 56.0% were female, 42.1% had low-level education, 41.8% had low salary (<¥3,000), and majority were married (93.4%). Most patients were in stage IV (81.3%), and 18.7% were in stage III. About 53.1% of the patients had tumor recurrence or progression, most type of progression was distant metastasis (57.2%). Majority (97.8%) were receiving medical treatment, 92.7% were under immunotherapy, 15.4% were under targeted therapy, and 1.1% were under chemotherapy (Table 1).

**TABLE 1:** Patient characteristics (*n* = 273).

Patient characteristics	No.	(%)
Age (years ± SD; range)	56.9 ± 13.2 (16–86)	
**Gender**		
Male	120	(44.0)
Female	153	(56.0)
**Marital status**		
Single	10	(3.7)
Married	255	(93.4)
Divorced/Widowed	8	(3.0)
**Education**		
Low	115	(42.1)
Middle	65	(23.8)
High	93	(34.1)
**Salary**		
<¥3,000	114	(41.8)
¥3,000–5,000	77	(28.2)
¥5,000–10,000	63	(23.1)
>¥10,000	19	(7.0)
Months after diagnosis (months ± SD; range)	21.6 ± 29.3 (1–196)	
**Cancer stage**		
Unresectable III	51	(18.7)
IV	222	(81.3)
**Disease progression**		
Yes	145	(53.1)
No	128	(46.9)
**Types of disease progression**		
Local	51	(35.2)
Distant	83	(57.2)
Local + distant	11	(7.6)
**Type of ongoing medical treatment**		
Chemotherapy	3	(1.1)
Targeted therapy	42	(15.4)
Immunotherapy	253	(92.7)
No	6	(2.2)

### Prevalence of Fear of Progression, Anxiety, and Depression

When using the FoP-Q-SF to detect FoP, the mean level of FoP was 39.9 ± 11.0, with 64.7% (174 patients) reporting elevated FoP. Using the STAI-6 to measure anxiety, the average level of anxiety was 13.1 ± 3.0, with 72.5% (198 patients) reporting elevated anxiety. Using the PHQ-9 to assess depressive symptoms, the mean score of depression was 6.4 ± 6.1, with 22.7% (62 patients) reporting elevated depression. 14.7% (40 patients) had concurrent FoP, anxiety and depression, 35.5% (97 patients) had concurrent FoP and anxiety, 2.2% (6 patients) had concurrent FoP and depression, 4.0% (11 patients) had concurrent anxiety and depression, 11.4% (31 patients) only had FoP, 18.3% (50 patients) only had anxiety, 1.8% (5 patients) only had elevated depression, and 12.1% (33 patients) did not report elevated FoP, anxiety and depression.

### Univariate Analysis

As shown in Table 2, high FoP was significantly associated with patient’s education (*P* = 0.022), disease duration (*P* = 0.009) and cancer recurrence or progression (*P* = 0.002). Patients who had low-level education, short disease duration and cancer progression (especially distant metastasis) tended to report high levels of FoP. Additionally, clinical anxiety was mainly associated with patient’s education (*P* = 0.004), salary (*P* = 0.024), cancer stage (*P* = 0.001), and cancer recurrence or progression (*P* < 0.001), those patients with low education level, low income, stage IV of cancer, and cancer progression (especially local recurrence and distant metastasis) had high levels of anxiety. Furthermore, depression scores were higher in patients who had short disease duration (*P* = 0.033) and cancer recurrence or progression (*P* = 0.018) in comparison with their counterparts. No significant differences in FoP, anxiety, or depression were observed for other variables such as age, gender, marital status, investigation time, and treatments.

**TABLE 2:** Factors associated with fear of progression, anxiety and depression.

	Fear of progression		FoP-Q-SF ≥ 34		Anxiety		STAI-6 ≥ 12		Depression		PHQ-9 ≥ 10	
						
	Mean ± SD	*P*	N	%	Mean ± SD	*P*	N	%	Mean ± SD	*P*	N	%
Age, y		0.424				0.796				0.692		
≤56	42.9 ± 11.1		71	61.2%	13.5 ± 3.1		84	72.4%	7.0 ± 6.3		28	24.1%
>56	42.9 ± 10.2		103	65.6%	13.5 ± 2.9		114	72.6%	7.1 ± 6.6		34	21.7%
Gender		0.804				0.773				0.914		
Male	39.7 ± 12.0		70	58.3%	13.2 ± 3.2		87	72.5%	6.2 ± 5.6		22	18.3%
Female	40.0 ± 10.2		104	68.0%	13.0 ± 2.9		111	72.5%	6.5 ± 6.5		40	26.1%
Marital status		0.421				0.306				0.230		
Single	41.5 ± 12.4		6	60.0%	13.7 ± 1.9		10	100.0%	5.1 ± 5.9		1	10.0%
Married	39.7 ± 11.0		162	63.5%	13.0 ± 3.1		181	71.0%	6.5 ± 6.1		60	23.5%
Divorced/Widowed	44.3 ± 9.7		6	75.0%	14.6 ± 2.3		7	87.5%	4.5 ± 7.3		1	12.5%
Education		0.022*				0.004**				0.562		
Low	41.3 ± 10.2		77	67.0%	13.6 ± 3.0		88	76.5%	6.5 ± 5.6		27	23.5%
Middle	41.1 ± 11.0		43	66.2%	13.4 ± 3.1		50	76.9%	6.4 ± 6.3		16	24.6%
High	37.3 ± 11.6		54	58.1%	12.3 ± 2.9		60	64.5%	6.1 ± 6.6		19	20.4%
Salary		0.265				0.024*				0.110		
<¥3,000	40.8 ± 10.1		76	66.7%	13.7 ± 3.0		90	78.9%	6.8 ± 6.1		29	25.4%
¥3,000–5,000	40.6 ± 10.5		51	66.2%	13.0 ± 2.9		57	74.0%	7.5 ± 7.4		25	32.5%
¥5,000–10,000	37.6 ± 12.0		36	57.1%	12.1 ± 3.1		38	60.3%	4.7 ± 4.0		5	7.9%
>¥10,000	38.6 ± 13.9		11	57.9%	12.8 ± 2.4		13	68.4%	4.6 ± 4.7		3	15.8%
Investigation time		0.720				0.351				0.704		
2020.02–2020.12	39.7 ± 11.0		71	61.2%	13.4 ± 2.9		90	77.6%	6.8 ± 6.8		28	24.1%
2021.01–2021.11	40.0 ± 11.0		103	65.6%	12.9 ± 3.2		108	68.8%	6.0 ± 5.5		34	21.7%
Months after diagnosis, m		0.009**				0.145				0.033*		
≤ 21	41.1 ± 10.6		132	70.2%	13.4 ± 3.0		141	75.0%	6.8 ± 6.2		46	24.5%
> 21	37.1 ± 11.5		42	49.4%	12.5 ± 3.1		57	67.1%	5.4 ± 5.8		16	18.8%
Cancer stage		0.104				0.001**				0.625		
Unresectable III	37.5 ± 10.3		31	60.8%	12.2 ± 2.0		34	66.7%	6.0 ± 5.8		12	23.5%
IV	40.4 ± 11.1		143	64.4%	13.3 ± 3.2		164	73.9%	6.4 ± 6.2		50	22.5%
Disease progression		0.002**				0.000***				0.018*		
Yes	41.8 ± 11.3		100	69.0%	14.5 ± 2.6		126	86.9%	7.2 ± 6.4		38	26.2%
No	37.7 ± 10.3		74	57.8%	11.4 ± 2.6		72	56.3%	5.4 ± 5.6		24	18.8%
Types of disease progression		0.002**				0.000***				0.054		
Local	37.5 ± 10.3		31	60.8%	12.1 ± 2.0		33	64.7%	5.9 ± 5.9		12	23.5%
Distant	44.5 ± 11.2		62	74.7%	15.7 ± 2.0		82	98.8%	8.3 ± 6.8		25	30.1%
Local + distant	42.0 ± 10.7		7	63.6%	16.7 ± 1.8		11	100.0%	4.6 ± 4.5		1	9.1%
Chemotherapy		0.064				0.801				0.286		
Yes	28.0 ± 6.2		3	100.0%	13.0 ± 1.7		3	100.0%	2.7 ± 2.3		0	0.0%
No	40.0 ± 11.0		174	64.4%	13.1 ± 3.1		195	72.2%	6.4 ± 6.1		62	23.0%
Target therapy		0.500				0.512				0.280		
Yes	39.0 ± 11.8		25	59.5%	13.0 ± 2.8		28	66.7%	7.4 ± 6.6		15	35.7%
No	40.0 ± 10.9		149	64.5%	13.1 ± 3.1		170	73.6%	6.2 ± 6.0		47	20.3%
Immunotherapy		0.131				0.055				0.561		
Yes	39.6 ± 10.8		160	63.2%	13.0 ± 3.0		181	71.5%	6.4 ± 6.1		57	22.5%
No	43.5 ± 12.4		14	70.0%	14.4 ± 2.8		17	85.0%	6.0 ± 6.6		5	25.0%

*FoP-Q-SF, Fear of Progression Questionnaire-Short Form; STAI-6, six-item short form of State Trait Anxiety Inventory; PHQ-9, nine-item short form of Patient Health Questionnaire.*

*P < 0.05; **P < 0.01; ***P < 0.001.

*Bold indicates P-value less than 0.05.*

### Multivariate Analysis

In multivariate logistic regression analysis, cancer recurrence or progression (*OR* = 1.960, *P* = 0.014 for FoP; *OR* = 23.235, *P* < 0.001 for anxiety; *OR* = 1.930, *P* = 0.035 for depression) was confirmed to be independently associated with higher FoP, anxiety and depression levels. Short disease duration (*OR* = 0.987, *P* = 0.006 for FoP; *OR* = 0.984, *P* = 0.030 for depression) was found to be independent factor of higher FoP and depression levels. Moreover, patients who were under stage IV of melanoma (*OR* = 14.394, *P* < 0.001) were more likely to report higher anxiety level (Table 3). The score of FoP was significantly positive correlated with anxiety (*r* = 0.466, *P* < 0.001) and depression (*r* = 0.382, *P* < 0.001). The score of anxiety and depression were found to be significantly positive correlated with each other (*r* = 0.309, *P* < 0.001).

**TABLE 3:** Multiple logistic regression for fear of progression, anxiety, and depression.

Variable	Exp (B)	95%CI lower	95%CI upper	*P*
**Fear of progression**				
**Education**				
Low	Ref			
Middle	1.046	0.539	2.030	0.894
High	0.724	0.405	1.294	0.276
Months after diagnosis (m)	0.987	0.978	0.996	0.006**
**Disease progression**				
No	Ref			
Yes	1.960	1.147	3.349	0.014*
**Anxiety**				
**Education**				
Low	Ref			
Middle	1.360	0.593	3.116	0.468
High	1.038	0.435	2.479	0.933
**Salary**				
<¥3,000	Ref			
¥3,000–5,000	0.834	0.376	1.850	0.655
¥5,000–10,000	0.466	0.183	1.184	0.108
>¥10,000	0.734	0.193	2.796	0.651
**Cancer stage**				
Unresectable III	Ref			
IV	14.394	3.929	52.730	0.000***
**Disease progression**				
No	Ref			
Yes	23.235	6.955	77.625	0.000***
**Depression**				
Months after diagnosis (m)	0.984	0.969	0.998	0.030*
**Disease progression**				
No	Ref			
Yes	1.930	1.047	3.561	0.035*

*P < 0.05; **P < 0.01; ***P < 0.001.

*Bold indicates P-value less than 0.05.*

## Discussion

The present study is one of the first cross-sectional studies to examine the psychological outcomes in patients with advanced melanoma during the COVID-19 pandemic. Our results showed that patients with advanced melanoma experienced high levels of FoP, anxiety and depression, which was persisting in the COVID-19 and post-COVID-19 era. We also found that FoP, anxiety, and depression were significantly positive correlated with each other, and disease duration, cancer stage, and cancer progression were independent predictors for these negative psychological symptoms in patients with advanced melanoma.

In these unselected inpatients with advanced melanoma, we found that 64.7% reported increased FoP, 72.5% experienced anxious symptoms, and 22.7% had elevated depression. Previous studies reported a wide variation of prevalence of psychological distress in melanoma patients, with the prevalence of FoP ranging from 0 to 77%, anxiety ranging from 15 to 49%, and depression ranging from 5 to 28% (29–37). Most studies focused on patients with early-stage melanoma, but only a few studies reported the psychological symptoms in patients with metastatic melanoma in small sample size (38–40). Recent researches demonstrate that the COVID-19 pandemic has induced increased levels of psychological distress among cancer patients. Wang et al. (41) showed that during the COVID-19 pandemic, 23.4% of Chinese cancer patients had depression, and 17.7% had anxiety. Frey et al. (42) also showed that 51.4% of cancer patients reported anxiety and 26.5% reported depression in the Unite State during the epidemics. Romito et al. (43) found that 36% of cancer patients had anxiety, 31% had depression during the first phase of the lockdown period in Italy. Chen et al. (44) found that 282 (86.5%) Chinese cancer patients reported FoP under the outbreak of COVID-19. Three other studies showed high levels of FoP in breast cancer patients with prevalence ranging from 17.2 to 84.1% (45–47). The highest prevalence of FoP was found in a recent study among hematological cancer patients showing that nearly all participants (127/134, 95%) reported clinical FoP (48). Until now, only one study assessed impact of COVID-19 on anxiety levels among cancer patients including 26 (8.5%) melanoma patients (49). Therefore, our study first implies that FoP, anxiety and depression in patients with advanced melanoma indeed aggravate during the COVID-19 pandemic.

Our multivariate analysis further confirms that disease duration, cancer stage and cancer progression are independent predictors for psychological symptoms in patients with advanced melanoma. Consistent with our results, Bell et al. (31) found evidence of high FoP levels in patients with new or recurrent melanoma. Hinnen et al. (29) reported that patients with melanoma of a higher stage were more likely to report elevated FoP scores. Wagner et al. (33) assessed demographic factors (e.g., women sex and being employed) associated with severity of FoP in patients with stage IA malignant melanoma. Moreover, previous researches showed that melanoma patients with advanced disease and short illness duration were more likely to report anxiety and depression (16, 36, 40, 50), which are similar to our results. Other sociodemographic factors (e.g., women, younger age, unmarried state, low education, and unemployment) were found to be strongly associated with high anxiety and depression in melanoma patients (16, 51). Numerous studies investigated factors associated with psychological distress in melanoma patients, however, very few studies used multivariate analysis to elucidate independent predictors of FoP, anxiety and depression. Our results from multivariate analysis highlight a need for paying close attention to the psychological distress of melanoma patients with short disease duration, cancer progression and advanced disease under the COVID-19 outbreak.

A key finding of our study is that we found that mental distress of patients with advanced melanoma did not diminish over time during the COVID-19 and post-COVID-19 era, since no significant differences in FoP, anxiety or depression were observed for investigation time in the present study. In addition, FoP, anxiety and depression were significantly positive correlated with each other in melanoma patients with advanced stage during the epidemic. These findings clearly emphasize a need of mental care of melanoma patients under the further attack of COVID-19 worldwide or future global health threats. Clinicians and psychologists urgently need to re-organize healthcare systems to offer essential medical and psychological services to melanoma patients throughout the COVID-19 pandemic. A high quality of psycho-oncological care may help people to better cope with cancer during the epidemic. Several interventions have been developed to address psychological distress of melanoma patients. A psychoeducational intervention comprised a psychoeducational booklet and three individual telephone-based psychotherapeutic sessions, which was effective to reduce FoP of early-stage melanoma patients with high-risk recurrence (30). A stepped-care model was an acceptable and feasible intervention to treat FoP in patients with metastatic melanoma, for those with subthreshold FoP were offered self-management, and for those with clinical FoP were provided with individual treatment (39). Consider high risk of person-to-person transmission of COVID-19, the current developments in non-contact intervention may be novel, safe and efficient ways of psychological care. Internet cognitive behavioral therapy, delivered online via a website and/or app, is used to provide information and support to cancer patients, survivors, and carers on managing unhelpful thinking and behaviors, normalizing feelings, and alleviating FoP, anxiety and depression (52–56). Moreover, Royce et al. (57) reported that most patients wished for a telemedical consultation, which might be a solution to facilitate patients’ access to health benefits and respect physical distancing. Our study and previous studies imply that it is necessary to proceed contact-free psychosocial education or cognitive behavioral therapy to reduce symptoms of FoP, anxiety and depression in patients with advanced melanoma and to guide people smoothly and safely through the epidemic.

The current study has several limitations. The first is that this study focused on Chinese cancer patients, findings cannot be generalized to other populations in other countries. Second, the cross-sectional design of this study made it impossible to compare these data with pre-pandemic distress status for the same cohort. To a certain extent, stratified analysis according to investigation time reflected the influence of different epidemic stage on the results, but it is still necessary to compare COVID-19 and post-COVID-19 era in patients with two interventions. Third, patients presented with advanced stage and recurrent disease, which probably led to higher baseline levels of distress of these patients. Increased mental distress was primarily collateral effects to the disease itself, which was exacerbated due to the impact of COVID-19 pandemic on medical management. Moreover, the current study did not explore the mental status of outpatients and caregivers. Future studies are needed to examine these factors and confirm these results.

The present research indicated that there was a large proportion of melanoma patients with high FoP, anxiety and depression in mainland China during the COVID-19 pandemic. Our study further explored the independent predictors of FoP, anxiety, and depression, such as illness duration, cancer stage, and disease progression. Particularly, these negative psychological symptoms of patients with advanced melanoma were positive correlated with each other and did not diminish over time in the COVID-19 and post-COVID-19 era. These findings emphasize the importance of developing psycho-oncological interventions targeting patients with advanced melanoma in the face of the epidemic in a longer run. Future study is needed to further examine the mental health problems, associated factors and effective interventions among patients with advanced melanoma during the COVID-19 pandemic.

## Conclusion

This study is one of the first to provide essential information about psycho-oncological needs of patients with advanced melanoma under the COVID-19 attack. Our study showed that high FoP, anxiety and depression were frequently reported problems among patients with advanced melanoma, which were significantly positive correlated with each other and persisting in the COVID-19 and post-COVID-19 era. Illness duration, cancer stage and disease progression were independent predictors for these negative psychological symptoms in patients with advanced melanoma. A better understanding of these findings could enable oncologists to develop and improve appropriate evidence-based psychological care for melanoma patients that targets particular symptoms during the ongoing COVID-19 pandemic.

## Data Availability Statement

The original contributions presented in the study are included in the article/supplementary material, further inquiries can be directed to the corresponding author.

## Ethics Statement

The patients/participants provided their written informed consent to participate in this study.

## Author Contributions

XW, ML, LZ, and H-MZ were responsible for study conception and design. XW, HJ, SK, and QS were responsible for acquisition of data. XW and ML were responsible for data analysis. XW, ML, HJ, SK, QS, and LZ were responsible for drafting. XW, ML, and H-MZ were responsible for revision of the manuscript. All authors approved the submitted version for publication.

## Conflict of Interest

The authors declare that the research was conducted in the absence of any commercial or financial relationships that could be construed as a potential conflict of interest.

## Publisher’s Note

All claims expressed in this article are solely those of the authors and do not necessarily represent those of their affiliated organizations, or those of the publisher, the editors and the reviewers. Any product that may be evaluated in this article, or claim that may be made by its manufacturer, is not guaranteed or endorsed by the publisher.

## Footnotes

1
www.wjx.cn
